# Machine learning early risk assessment model for acute kidney injury in critically ill children: a retrospective cohort study

**DOI:** 10.3389/fped.2026.1847661

**Published:** 2026-07-09

**Authors:** Linyao Xie, Chao Chen, Chaojie Zhang, Lizhi Chen, Yijuan Li

**Affiliations:** 1 Department of Pediatrics, The First Affiliated Hospital, Sun Yat-sen University, Guangzhou, China; 2 Department of Pediatrics, The Seventh Affiliated Hospital, Sun Yat-sen University, Shenzhen, China

**Keywords:** acute kidney injury, critically ill children, early risk assessment model, machine learning, pediatric intensive care database

## Abstract

**Background:**

Acute kidney injury (AKI) is a common severe complication in intensive care unit (ICU). However, an early risk assessment model that can accurately and promptly predict the risk of AKI in critically ill children remains lacking.

**Methods:**

This retrospective study included 3,799 children from the Pediatric Intensive Care (PIC) database. The dataset was randomly divided into training set and validation set at a ratio of 7:3. LASSO regression and the Boruta algorithm were employed for feature selection, and the selected variables were incorporated into five machine learning models (Logistic Regression, Random Forest, XGBoost, LightGBM, Support Vector Machine) for training and construction. Model performance was evaluated using the area under the receiver operating characteristic curve (AUC), and the SHAP framework was applied for interpretability analysis of the optimal model.

**Results:**

On the validation set, the XGBoost model demonstrated the best risk stratification performance among all five algorithms. SHAP analysis identified bicarbonate, magnesium, activated partial thromboplastin time, lymphocyte count, and thrombin time as the five most important features contributing to the model's predictions.

**Conclusion:**

We successfully developed an AKI risk stratification model based on early available clinical data. The model demonstrated acceptable discriminative ability and clinical interpretability in critically ill children, offering potential support for early intervention and improving prognosis.

## 1 Background

Acute kidney injury (AKI) is a common and serious complication among critically ill children and poses a significant threat to survival and prognosis (1, 2). Epidemiological data indicate that the incidence of AKI in children in the intensive care unit (ICU) is approximately 30% to 50%, and it is closely associated with increased mortality, prolonged hospital stays, and long-term renal impairment (3–5). Despite significant advancements in critical care technology in recent years, early identification and intervention for AKI remain challenging in clinical practice (6). Of particular concern is that AKI often has an insidious onset and rapid progression; if not diagnosed and treated promptly, it can easily lead to multiple organ dysfunction, severely affecting the quality of life and survival of children (7). Therefore, timely and accurate early risk assessment for AKI in critically ill children is of paramount importance for guiding early clinical intervention and improving patient outcomes.

In recent years, the application of machine learning in medicine has become increasingly widespread, demonstrating powerful capabilities in data mining and pattern recognition (8). By integrating multi-dimensional data including vital signs, laboratory tests, and treatments, machine learning techniques hold promise for enabling the early and precise identification of AKI risk. Several studies have successfully applied various machine learning algorithms to predict the risk of AKI in critically ill patients with sepsis, post-cardiac surgery, and acute respiratory distress syndrome (9–12). For example, Deng et al. compared the performance of the logistic XGBoost model for predicting the prognosis in hospitalized children with AKI (13). Sandokji et al. used multiple machine learning algorithms to construct a time-updated prediction model to accurately predict the impending AKI in hospitalized children (14). Song et al. developed a machine learning model for postoperative AKI following cardiac surgery, achieving an accuracy of 0.72 (11). Although these models show acceptable to good discrimination ability, they usually cannot provide in-depth insights into how individual predictive factors at the patient level affect risk prediction. Moreover, systematic research on AKI risk prediction models for the general critically ill pediatric population remains relatively scarce, and few studies have focused on model interpretability.

To address this gap, this study introduces and optimizes machine learning algorithms to build an AKI risk stratification model using early available clinical data for critically ill children, with a particular emphasis on model interpretability. Our main innovation lies in the application of SHAP (Shapley Additive Explanations) analysis, which decomposes model predictions and visualizes the contribution of each feature at both the group and individual levels. We hypothesize that by adopting effective feature selection methods to identify key predictive variables and using SHAP for interpretation, a model with acceptable risk assessment accuracy and clinical actionable insights could be constructed. The aim of this study is to develop a clinically interpretable machine learning system for early-stage AKI risk assessment in critically ill Chinese children. Our findings are expected to provide a novel approach for early warning of AKI, revealing how specific clinical variables drive risk, and thereby promoting the application of interpretable machine learning in pediatric critical care research.

## 2 Materials and methods

### 2.1 Study data

We conducted a retrospective analysis using the Pediatric Intensive Care (PIC) database. PIC is a large, pediatric-specific, single-center, bilingual database containing information on children admitted to the ICU at the Children's Hospital, Zhejiang University School of Medicine, China, between 2,010 and 2,018 (15). The PIC database includes vital sign measurements, medications, laboratory measurements, fluid balance, diagnostic codes, length of stay, and survival data. The PIC database is publicly available. This project was approved by the Institutional Review Board/Ethics Committee of the Children's Hospital, Zhejiang University School of Medicine (2019 IRB 052). As the project did not affect clinical care and all protected health information was de-identified, the requirement for informed consent was waived. We formally applied for access through the procedures documented on the PIC website and PhysioNet, signing a data use agreement. We handled the data responsibly, adhering to the principles of collaborative research.

**Figure 1:**
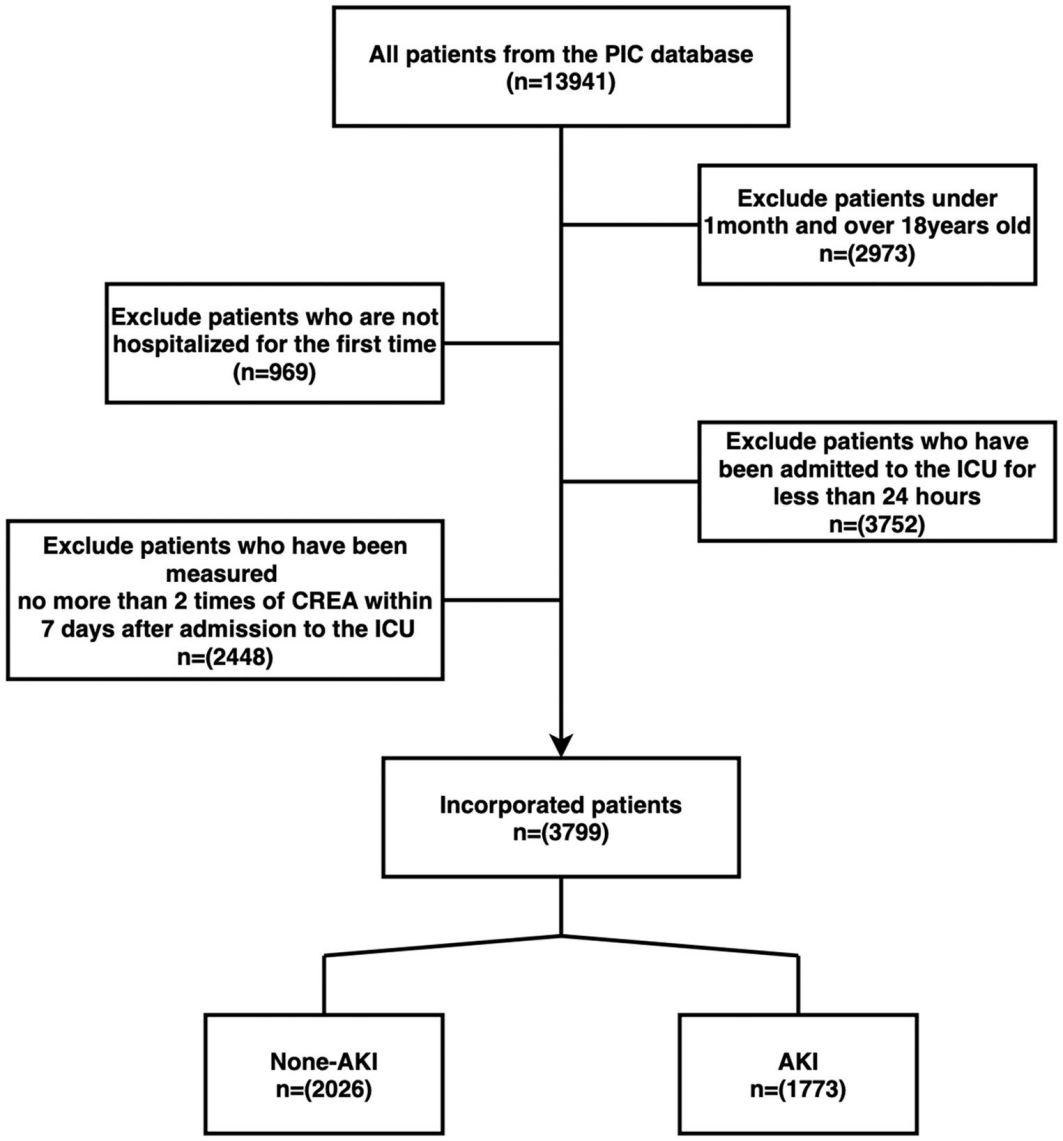
Flowchart of children admitted to the ICU and enrolled in the study.

Inclusion criteria were: (1) age 1 month to 18 years; and (2) first admission to the ICU. Exclusion criteria were: (1) ICU stay less than 24 h; and (2) fewer than 2 creatinine measurements within 7 days of ICU admission (Figure 1).

### 2.2 Study variables

Based on the existing literature, we first identified potential risk factors for pediatric AKI, including demographic characteristics (age, sex), vital signs (blood pressure, respiratory rate, heart rate), clinical severity indicators (e.g., vasopressor use), and laboratory parameters (electrolytes, liver function tests, blood gas indicators) (14, 16). Next, we selected predictors with clinical plausibility, some of which have biological or physiological mechanisms that could explain the pathogenesis of AKI, such as inflammatory and coagulation indicators (17, 18). Additionally, primary diagnoses and comorbidities (sepsis, congenital heart disease, chronic kidney disease, and tumors) were extracted. Finally, data availability in the electronic medical record system determined the final set of included variables; after evaluating missing data, variables with a missing rate exceeding 20% were excluded. The final set of variables is shown in Table 1.

**Table 1:** Detailed definitions of variables.

Variables	Full name	Unit	Type	Description
**Age**	Age	Year	Continuous	Age of the patient
**Gender**	Gender	-	Categorial	Male/Female
**Primary disease**	Primary disease	-	Categorial	Cardiovascular/Respiratory/Gastrointestinal/ Neurological/Urogenital/Others
**Heart Rate**	Heart Rate	Bmp	Continuous	First value on day 1 in ICU
**Respiratory Rate**	Respiratory Rate	Insp/min	Continuous	First value on day 1 in ICU
**RBC**	Red blood cell	×10^9/L	Continuous	First value on day 1 in ICU
**WBC**	White blood cell	×10^9/L	Continuous	First value on day 1 in ICU
**PLT**	Platelets	×10^9/L	Continuous	First value on day 1 in ICU
**Hb**	Hemoglobin	g/L	Continuous	First value on day 1 in ICU
**RDW**	Red Blood Cell Distribution Width	%	Continuous	First value on day 1 in ICU
**NEUT**	Neutrophils	×10^9/L	Continuous	First value on day 1 in ICU
**LYMPH**	Lymphocyte	×10^9/L	Continuous	First value on day 1 in ICU
**TBIL**	Total Bilirubin	μmol/L	Continuous	First value on day 1 in ICU
**ALB**	Albumin	g/L	Continuous	First value on day 1 in ICU
**Ca**	Calcium	mmol/L	Continuous	First value on day 1 in ICU
**Na**	Sodium	mmol/L	Continuous	First value on day 1 in ICU
**Mg**	Magnesium	mmol/L	Continuous	First value on day 1 in ICU
**K**	Potassium	mmol/L	Continuous	First value on day 1 in ICU
**AG**	Anion gap	mmol/L	Continuous	First value on day 1 in ICU
**Bicarbonate**	bicarbonate	mmol/L	Continuous	First value on day 1 in ICU
**CRP**	C-Reactive Protein	mg/L	Continuous	First value on day 1 in ICU
**Lac**	Lactic acid	mmol/L	Continuous	First value on day 1 in ICU
**GLU**	Glucose	mmol/L	Continuous	First value on day 1 in ICU
**D-Dimer**	D-Dimer	mg/L	Continuous	First value on day 1 in ICU
**Fib**	Fibrinogen	g/L	Continuous	First value on day 1 in ICU
**INR**	International Normalized Ratio	-	Continuous	First value on day 1 in ICU
**PT**	Prothrombin Time	Sec	Continuous	First value on day 1 in ICU
**TT**	Thrombin Time	Sec	Continuous	First value on day 1 in ICU
**APTT**	Activated Partial Thromboplastin Time	Sec	Continuous	First value on day 1 in ICU
**CHD**	Congenital heart disease	-	Categorial	1/0
**CKD**	Chronic kidney disease	-	Categorial	1/0
**Tumor**	Tumor	-	Categorial	1/0
**Sepsis**	Sepsis	-	Categorial	1/0
**Used vasopressor**	Used vasopressor	-	Categorial	1/0，used within 24 h in ICU

### 2.3 AKI diagnostic criteria

AKI was defined according to the Kidney Disease Improving Global Outcomes (KDIGO) criteria as a 50% increase in the lowest creatinine level within 7 days or an increase in creatinine level of 0.3 mg/dL within 48 h after ICU admission (19, 20).

### 2.4 Variable selection

#### 2.4.1 LASSO regression

LASSO regression performs feature selection in high-dimensional data by imposing an L1 penalty on regression coefficients, shrinking the coefficients of unimportant variables to zero and thereby effectively preventing model overfitting. The analysis was performed using the glmnet package in R. For the binary outcome, the family was set to “binomial”. The penalty parameter *α* was set to 1 for LASSO regression. The optimal penalty coefficient *λ* was determined via 10-fold cross-validation. To prioritize model simplicity and generalizability, we selected the *λ* value within one standard error of the minimum cross-validation error (lambda.1se). Variables with non-zero coefficients at this *λ* value were retained.

#### 2.4.2 Boruta algorithm

To comprehensively evaluate the importance of predictor variables, we employed the Boruta feature selection algorithm. This algorithm, based on the Random Forest framework, selects features by comparing the importance of original variables with that of randomly shuffled shadow variables. Categorical variables were factorized, and the response variable was converted to a factor type. The dataset used for Boruta contained no missing values after multiple imputation, so no listwise deletion was performed.

### 2.5 Machine learning algorithms

#### 2.5.1 Logistic regression with elastic net regularization

We employed a logistic regression model with elastic net regularization, which introduces both L1 (LASSO) and L2 (Ridge) penalty terms into the loss function, balancing feature selection and model stability. The regularization mixing parameter *α* was set to 0.5, and the optimal penalty coefficient *λ* was determined via 5-fold cross-validation. This approach prevents overfitting while retaining good model interpretability, as the regression coefficients intuitively reflect the direction and magnitude of each predictor's influence on AKI risk.

#### 2.5.2 Random forest

We applied the Random Forest ensemble learning algorithm, which enhances model generalizability by constructing multiple decision trees and aggregating their predictions. Model parameters were set to generate 200 trees, with a minimum of 5 samples per node for splitting. Stratified and balanced sampling strategies were adopted to address class imbalance. This algorithm is insensitive to outliers and automatically assesses variable importance, facilitating subsequent feature analysis.

#### 2.5.3 XGBoost (extreme gradient boosting)

XGBoost is an advanced gradient boosting decision tree algorithm that iteratively trains a series of decision trees, with each new tree aiming to correct the residuals of the previous ones. To prevent overfitting, we employed a strong regularization strategy, including limiting the maximum tree depth to 4 layers, setting a low learning rate (0.05), and introducing L1 and L2 regularization terms. Additionally, the scale_pos_weight parameter was used to adjust sample weights to handle class imbalance, enabling the model to capture complex nonlinear relationships.

#### 2.5.4 LightGBM (light gradient boosting machine)

LightGBM is a gradient boosting framework based on a histogram algorithm designed to increase training speed and reduce memory consumption. We set the number of leaves to 20, maximum depth to 5 layers, and learning rate to 0.05. To handle class imbalance, we directly assigned a weight of 2 to each AKI-positive sample instead of using built-in weight parameters. This algorithm significantly improves computational efficiency on large-scale data while maintaining prediction accuracy.

#### 2.5.5 Support vector machine (SVM)

A Support Vector Machine with a radial basis function kernel was trained on the full training set after feature scaling (centering and scaling). Hyperparameters (cost C and gamma) were tuned via grid search, with search ranges of C ∈ {0.1, 0.5, 1, 2, 5} and gamma ∈ {0.01, 0.05, 0.1, 0.5, 1}. The final model used the combination that maximized the cross-validated AUC. Class imbalance was addressed by assigning a higher weight to AKI-positive samples. The model was implemented using the svm function from the e1071 package with probability predictions enabled.

#### 2.5.6 Hyperparameter tuning

All machine learning models were optimized using a grid search strategy with 5-fold cross-validation on the training set. The hyperparameter search ranges for each algorithm are summarized in Supplementary Table 4. The optimal combination for each model was selected based on the highest cross-validated AUC. A fixed random seed (123) was used for all randomized procedures to ensure reproducibility. The final hyperparameters for each model are listed in Supplementary Table 5.

### 2.6 SHAP interpretability analysis

To gain a deeper understanding of the risk assessment mechanism of the optimal machine learning model and to enhance its clinical interpretability, we employed the SHAP analysis framework. Based on cooperative game theory, SHAP assigns an importance value to each feature for each prediction instance, quantifying the contribution of that feature to the model's output. Specifically, we calculated the mean absolute SHAP value for each feature as a global importance indicator and analyzed the local influence patterns of features in individual samples.

### 2.7 Statistical analysis

Data were retrieved and extracted from the database using PostgreSQL (V.17.0) and Navicat Premium (V.17.0). The extracted data were subsequently cleaned and preprocessed in the R environment (version 4.5.1). Variables with more than 20% missing values were removed (Supplementary Table 1). Multiple imputation for missing data was performed using the “mice” package. Specifically, we generated 5 imputed datasets (m = 5), applying Predictive Mean Matching for all continuous variables and polytomous regression for categorical variables. A sensitivity analysis was conducted to ensure the quality of the imputed data. The distribution of continuous variables was assessed using Q-Q plots. For continuous variables in the complete dataset, data conforming to a normal distribution were presented as mean ± standard deviation, and intergroup comparisons were performed using the independent samples t-test; non-normally distributed data were presented as median (interquartile range), and intergroup comparisons were performed using the Mann–Whitney U test. Categorical variables were presented as frequency (percentage), and intergroup comparisons were performed using the Chi-square test or Fisher's exact test, as appropriate. To account for multiple hypothesis testing in baseline comparisons, we applied the Bonferroni correction to all *p*-values.

This study adopted a systematic data splitting strategy, randomly dividing the total dataset into the training and the validation sets at a 7:3 ratio, and we verified the comparability of baseline characteristics between the two sets. In the training set, all variables were included in the LASSO regression and Boruta algorithms for feature selection; the common variables selected by both methods were then chosen. The final optimized variable set was incorporated into five machine learning models (Logistic Regression, Random Forest, XGBoost, LightGBM, and SVM). Models were constructed through hyperparameter tuning, and their risk stratification performances were evaluated and compared on the validation set. Model performance was comprehensively compared using a unified set of evaluation metrics, including accuracy, sensitivity, specificity, area under the receiver operating characteristic curve (AUC), decision curve analysis (DCA), and calibration curves. To further explain the decision-making mechanism of the optimal model, SHAP analysis was used to quantify the contribution of each feature to individual predictions, revealing the global importance of key predictor variables and their complex nonlinear relationships with AKI risk. The statistical significance level was set at two-sided *P* < 0.05.

## 3 Results

### 3.1 Baseline characteristics

A total of 3,799 patients were included in this study, of whom 1,773 (46.6%) developed AKI during hospitalization. There were no significant differences in baseline characteristics before and after data imputation (Supplementary Table 2). Comparison of baseline characteristics between the AKI and non-AKI groups is shown in Table 2.

**Table 2:** Baseline characteristics of the AKI and non-AKI groups.

Variable	Overall *N* = 3,799^a^	0 *N* = 2,026^a^	1 *N* = 1,773^a^	P	*P*-Bonferroni
**Age**	1.46 (0.49, 4.73)	1.42 (0.51, 4.43)	1.50 (0.46, 4.99)	0.539	1.000
**RBC**	3.75 (3.27, 4.25)	3.75 (3.29, 4.22)	3.75 (3.24, 4.29)	0.723	1.000
**WBC**	9.28 (6.27, 13.58)	9.21 (6.41, 13.10)	9.41 (6.07, 14.24)	<0.001	0.010
**PLT**	211.00 (135.00, 323.00)	215.00 (147.00, 319.00)	207.00 (120.00, 328.00)	0.091	1.000
**Hb**	103.00 (91.00, 117.00)	103.00 (91.00, 116.00)	104.00 (91.00, 118.00)	0.203	1.000
**RDW**	13.90 (13.00, 15.30)	13.70 (12.80, 15.00)	14.10 (13.20, 15.50)	<0.001	0.002
**NEUT**	5.94 (3.41, 9.73)	6.00 (3.56, 9.58)	5.85 (3.26, 10.06)	0.146	1.000
**LYMPH**	2.13 (1.30, 3.24)	2.18 (1.42, 3.14)	2.07 (1.19, 3.32)	0.726	1.000
**TBIL**	10.80 (6.50, 18.60)	11.10 (6.70, 18.10)	10.50 (6.20, 19.40)	<0.001	0.020
**ALB**	37.90 (33.80, 41.40)	38.10 (34.50, 41.40)	37.50 (32.80, 41.60)	<0.001	0.002
**Ca**	2.23 (2.10, 2.35)	2.25 (2.13, 2.35)	2.21 (2.06, 2.34)	<0.001	<0.001
**Na**	138.00 (135.00, 141.00)	138.00 (135.00, 141.00)	138.00 (134.00, 141.00)	0.003	0.082
**Mg**	0.86 (0.80, 0.93)	0.86 (0.79, 0.92)	0.87 (0.81, 0.95)	<0.001	<0.001
**K**	3.60 (3.20, 4.00)	3.60 (3.20, 4.00)	3.60 (3.30, 4.10)	<0.001	<0.001
**Bicarbonate**	22.60 (20.40, 24.30)	22.90 (21.20, 24.50)	22.10 (19.40, 24.00)	<0.001	<0.001
**CRP**	18.00 (4.00, 49.00)	19.49 (3.10, 50.00)	15.00 (4.00, 48.10)	0.423	1.000
**Lac**	1.90 (1.30, 2.80)	1.80 (1.30, 2.60)	2.10 (1.30, 3.30)	<0.001	<0.001
**GLU**	7.60 (5.90, 10.20)	7.70 (6.00, 10.02)	7.30 (5.70, 10.60)	0.093	1.000
**D-Dimer**	1.62 (0.87, 3.45)	1.45 (0.83, 2.59)	1.92 (0.93, 4.66)	<0.001	<0.001
**Fib**	2.13 (1.56, 2.85)	2.22 (1.68, 2.95)	2.01 (1.42, 2.72)	<0.001	<0.001
**INR**	1.14 (1.01, 1.33)	1.13 (1.01, 1.30)	1.16 (1.02, 1.39)	<0.001	<0.001
**PT**	13.70 (12.10, 16.00)	13.60 (12.10, 15.60)	13.90 (12.20, 16.70)	<0.001	<0.001
**TT**	19.00 (16.90, 22.00)	18.46 (16.50, 21.04)	19.80 (17.40, 23.10)	<0.001	<0.001
**APTT**	34.80 (28.80, 46.60)	33.50 (28.42, 42.30)	36.90 (29.10, 51.10)	<0.001	<0.001
**Gender**				0.123	1.000
F	1,680.0 (44.2%)	920.0 (45.4%)	760.0 (42.9%)		
M	2,119.0 (55.8%)	1,106.0 (54.6%)	1,013.0 (57.1%)		
**CHD**				<0.001	0.002
0	3,593.0 (94.6%)	1,888.0 (93.2%)	1,705.0 (96.2%)		
1	206.0 (5.4%)	138.0 (6.8%)	68.0 (3.8%)		
**CKD**				<0.001	<0.001
0	3,777.0 (99.4%)	2,026.0 (100.0%)	1,751.0 (98.8%)		
1	22.0 (0.6%)	0.0 (0.0%)	22.0 (1.2%)		
**Tumor**				0.029	0.884
0	3,472.0 (91.4%)	1,871.0 (92.3%)	1,601.0 (90.3%)		
1	327.0 (8.6%)	155.0 (7.7%)	172.0 (9.7%)		
**Sepsis**				0.034	1.000
0	3,673.0 (96.7%)	1,971.0 (97.3%)	1,702.0 (96.0%)		
1	126.0 (3.3%)	55.0 (2.7%)	71.0 (4.0%)		
**Used vasopressor**				<0.001	<0.001
0	2,881.0 (75.8%)	1,344.0 (66.3%)	1,537.0 (86.7%)		
1	918.0 (24.2%)	682.0 (33.7%)	236.0 (13.3%)		
**Primary disease**				<0.001	<0.001
Cardiovascular	1,272.0 (33.5%)	804.0 (39.7%)	468.0 (26.4%)		
Gastrointestinal	305.0 (8.0%)	153.0 (7.6%)	152.0 (8.6%)		
Hematologic	247.0 (6.5%)	106.0 (5.2%)	141.0 (8.0%)		
Neurological	588.0 (15.5%)	327.0 (16.1%)	261.0 (14.7%)		
Others	714.0 (18.8%)	316.0 (15.6%)	398.0 (22.4%)		
Respiratory	573.0 (15.1%)	289.0 (14.3%)	284.0 (16.0%)		
Urogenital	100.0 (2.6%)	31.0 (1.5%)	69.0 (3.9%)		

a Median (Q1, Q3); *n* (%).

### 3.2 Predictor selection

**Figure 2:**
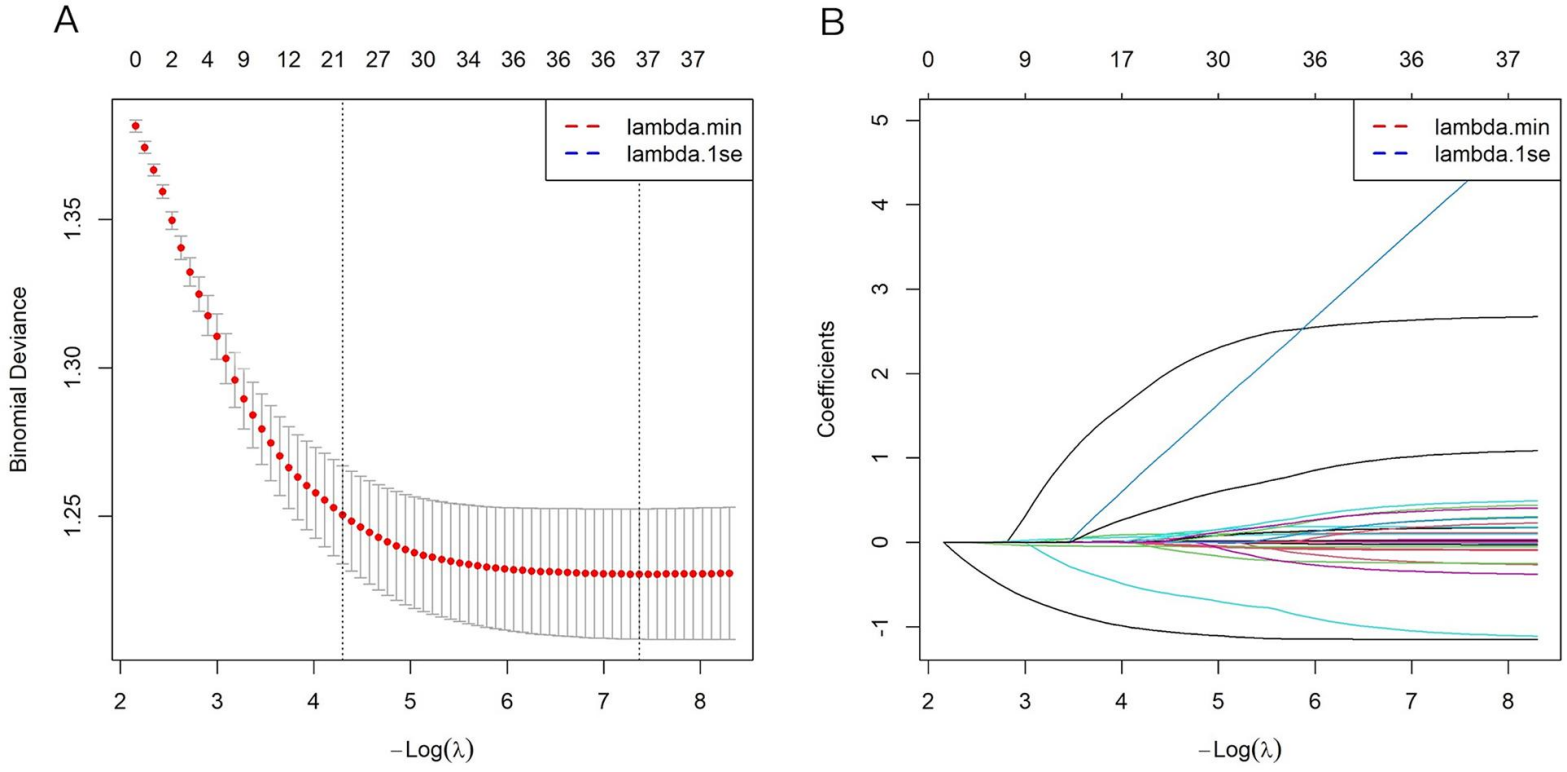
LASSO regression results for feature selection. **(A)** Cross-validation deviance curve as a function of log(λ). The left vertical dashed line indicates lambda.1se—the largest λ value within one standard error of the minimum cross-validated deviance, which yields the most parsimonious model (selected for this study to prioritize model simplicity and generalizability). The right vertical dashed line indicates lambda.min—the λ value with the lowest cross-validated deviance. Error bars represent ± 1 standard deviation of the cross-validated deviance. **(B)** Coefficient shrinkage trajectories for all predictor variables as the L1 penalty (log λ) increases. Each colored line represents a predictor variable; coefficients of less important variables progressively shrink toward zero as *λ* increases, while important variables retain non-zero coefficients.

**Figure 3:**
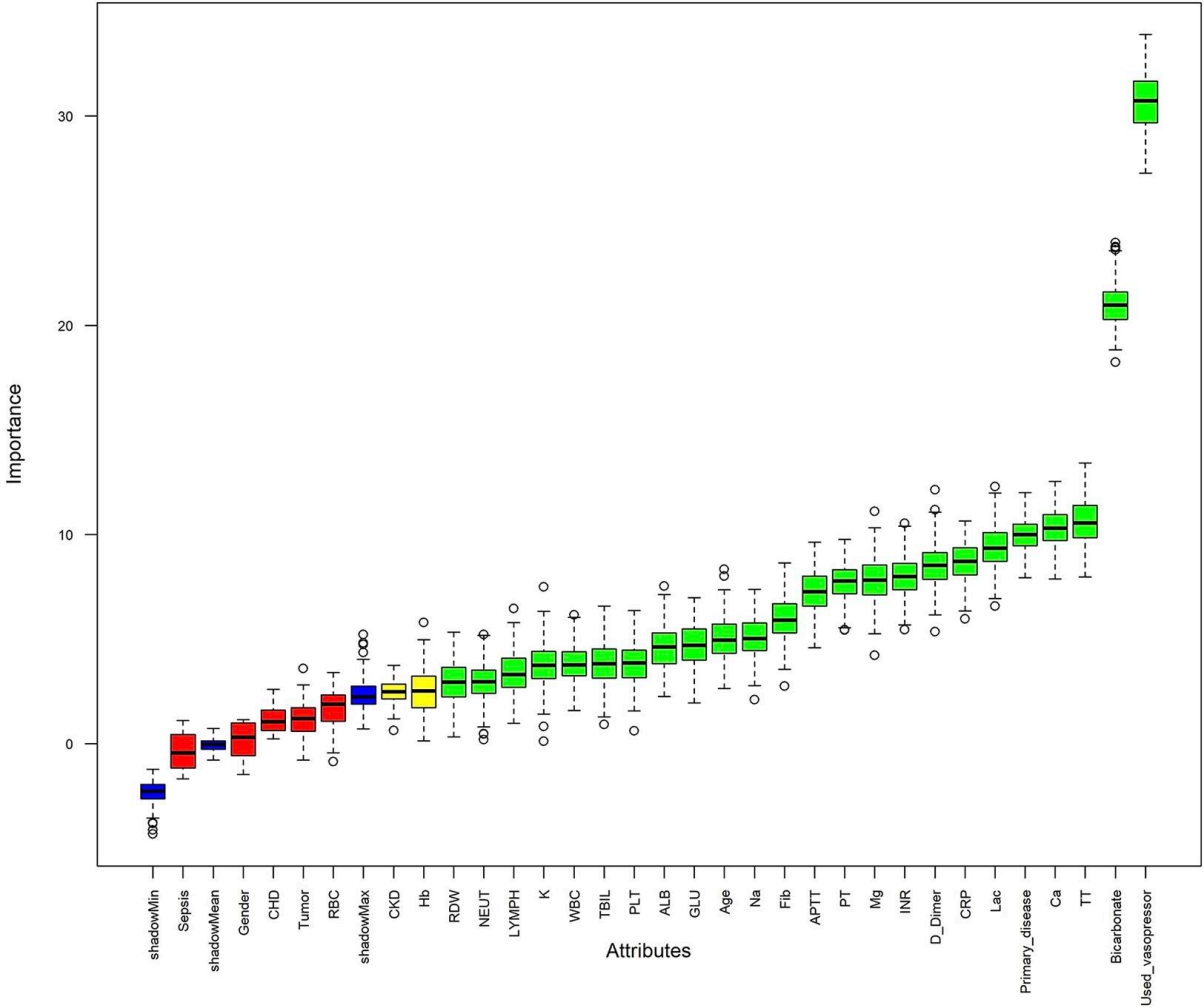
Boruta algorithm feature selection results. The algorithm compares each original variable's importance score (Z-score from Random Forest) with shadow variables (randomly permuted copies). Green boxes: Confirmed features—variables whose importance significantly exceeds the maximum shadow importance (selected for the final model). Yellow boxes: Tentative features—variables with borderline importance that did not reach the confirmation threshold. Red boxes: Rejected features—variables whose importance did not exceed shadow variables. Blue boxes: Shadow variables used as the reference for importance comparison.

The 3,799 patients were divided into training group and validation group in a 7:3 ratio. Statistical analysis showed no significant difference between the training group and the validation group. In this study, LASSO regression identified 19 feature factors (Figure 2). Simultaneously, the Boruta algorithm identified 24 key factors by accurately estimating the importance of each feature (Figure 3). By comparing the results obtained from LASSO regression and the Boruta algorithm, we selected the same set of feature variables chosen by both methods. A total of 14 variables were selected for model construction.

### 3.3 Model performance comparison

**Figure 4:**
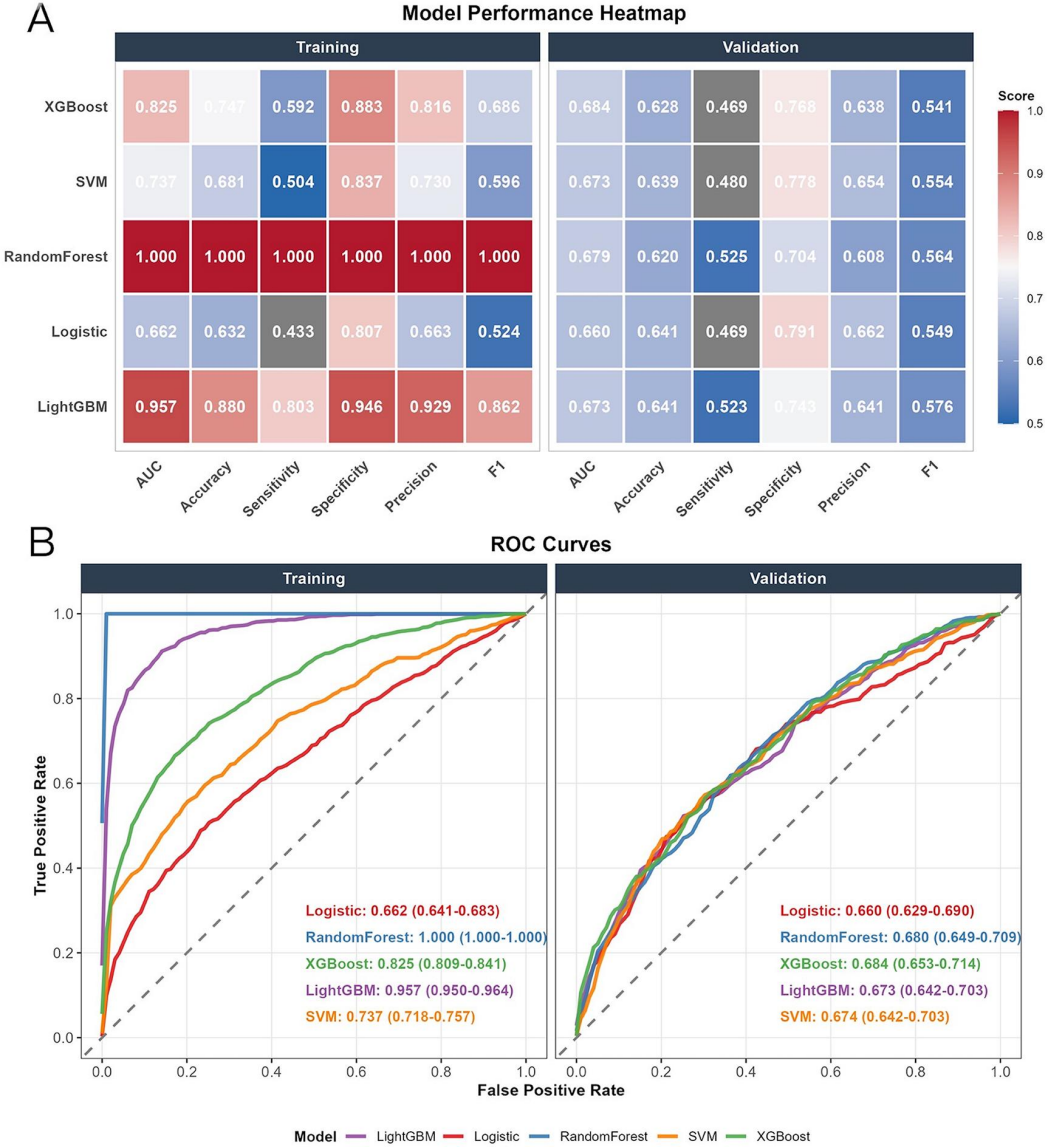
Performance and comparison of five different risk stratification models. (A) Comparison of performance metrics between training and validation sets; (B) Comparison of ROC curve between training and validation sets.

The model performance comparison revealed notable discrepancies between the training and validation sets (Figure 4). While ensemble methods achieved high discriminative ability on the training set, varying degrees of overfitting were observed. On the training set, Random Forest achieved perfect performance (AUC = 1.000), followed by LightGBM (AUC = 0.9571) and XGBoost (AUC = 0.8247); the training AUCs for Support Vector Machine and Logistic Regression were 0.7371 and 0.6616, respectively. On the validation set, however, all models demonstrated comparable AUC values that were significantly lower: XGBoost achieved the highest validation AUC of 0.6838, followed by Random Forest (0.6795), LightGBM (0.6728), Support Vector Machine (0.6726), and Logistic Regression (0.6599). The marked decline from near-perfect training AUCs to approximately 0.67–0.68 for Random Forest and LightGBM indicates a substantial risk of overfitting, whereas Logistic Regression showed the smallest performance decrease (training AUC 0.6616 vs. validation AUC 0.6599), suggesting greater stability.

The results of repeated cross-validation further confirm these observations (Supplementary Figure 1 and Supplementary Table 6). The average AUC (including standard deviation) of 5-fold cross-validation were: XGBoost 0.6749 (SD = 0.0229), Random Forest 0.6748 (SD = 0.0222), Support Vector Machine 0.6704 (SD = 0.0219), LightGBM 0.6699 (SD = 0.0201), and Logistic Regression 0.6525 (SD = 0.0193). The narrow standard deviation indicates that all models performed stably across each fold. Calibration metrics are summarized in Supplementary Table 6. XGBoost achieved the lowest Brier score (0.2216) and a non-significant Hosmer-Lemeshow test (*p* = 0.2243), indicating good overall calibration. Its calibration slope was 0.8789, suggesting a slight overfitting. Taken together, these two metrics indicate that while the model's overall calibration is acceptable, its predicted probabilities should be interpreted with caution. Recalibration techniques may further improve the model's clinical utility in future applications. Logistic regression, despite a calibration curve visually closer to the diagonal, showed a significant HL test (*p* = 0.0207), suggesting systematic miscalibration. The DCA curve showed that the XGBoost model provided positive net benefit compared with ‘treat all’ and ‘treat none’ strategies for threshold probabilities ranging from approximately 0.1 to 0.4, indicating potential clinical utility as a risk screening tool despite its modest AUC (Supplementary Figure 2). Therefore, XGBoost offers a reasonable balance between discrimination and calibration.

### 3.4 SHAP analysis

**Figure 5:**
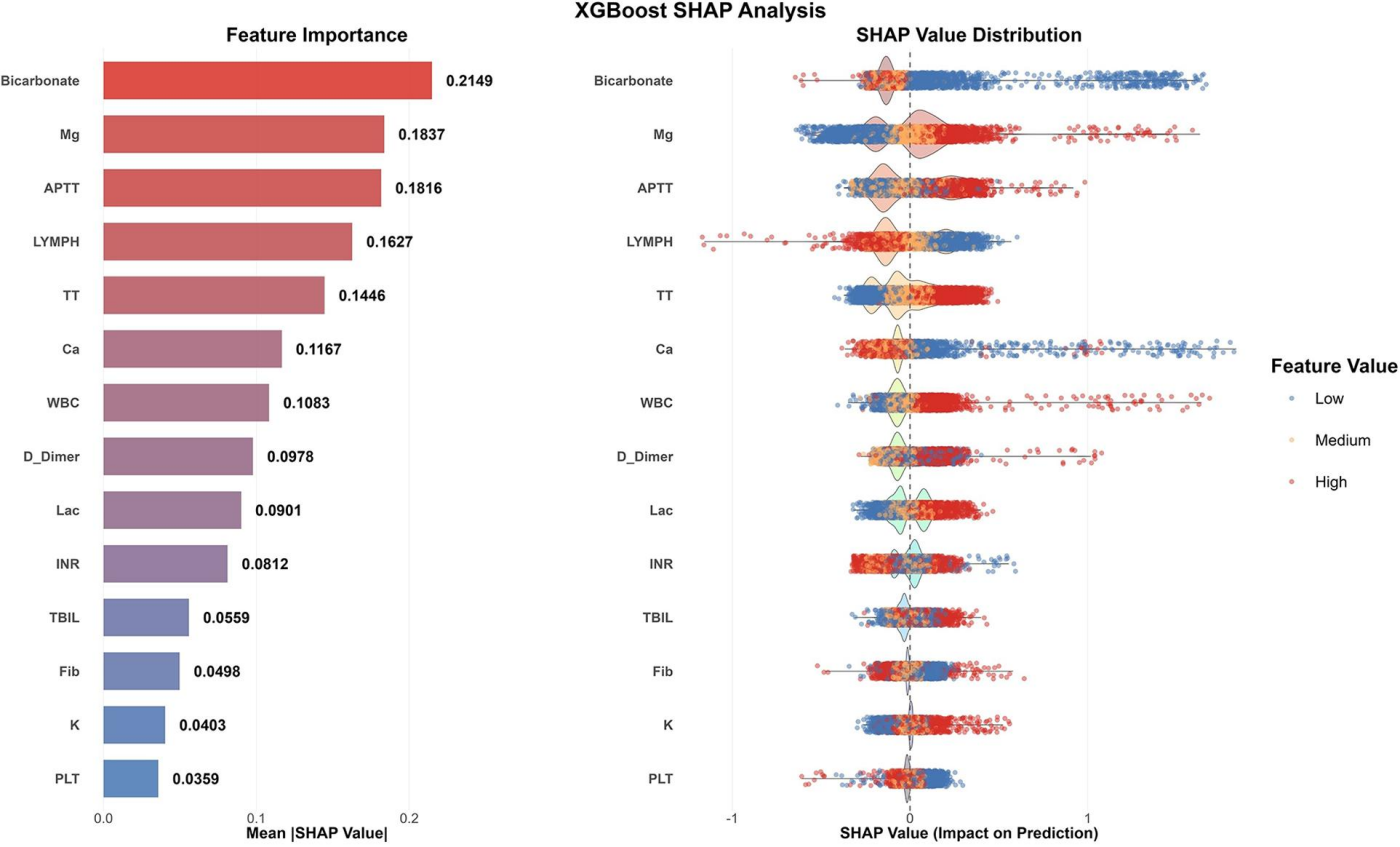
Feature importance ranking plot and SHAP summary plot for the XGBoost model.

Based on the best-performing XGBoost model, we used SHAP values to interpret the risk assessment results, quantifying the contribution of each feature to AKI risk prediction (Figure 5). The analysis revealed a distinct gradient in feature importance, with acid-base balance, electrolyte status, and coagulation function indicators dominating the predictions. Among them, bicarbonate had the highest mean absolute SHAP importance, accounting for 13.74% of the total feature importance, followed by magnesium (Mg, 11.75%), activated partial thromboplastin time (APTT, 11.62%), lymphocyte count (LYMPH, 10.41%), and thrombin time (TT, 9.25%). The top five features cumulatively contributed 56.77% of the overall importance, indicating that acid-base balance, coagulation function, and immune/inflammatory status play critical roles in the development of AKI. The top ten features (bicarbonate, Mg, APTT, LYMPH, TT, calcium, white blood cell count, D-dimer, lactate, and INR) cumulatively accounted for 88.37% of the total importance.

**Figure 6:**
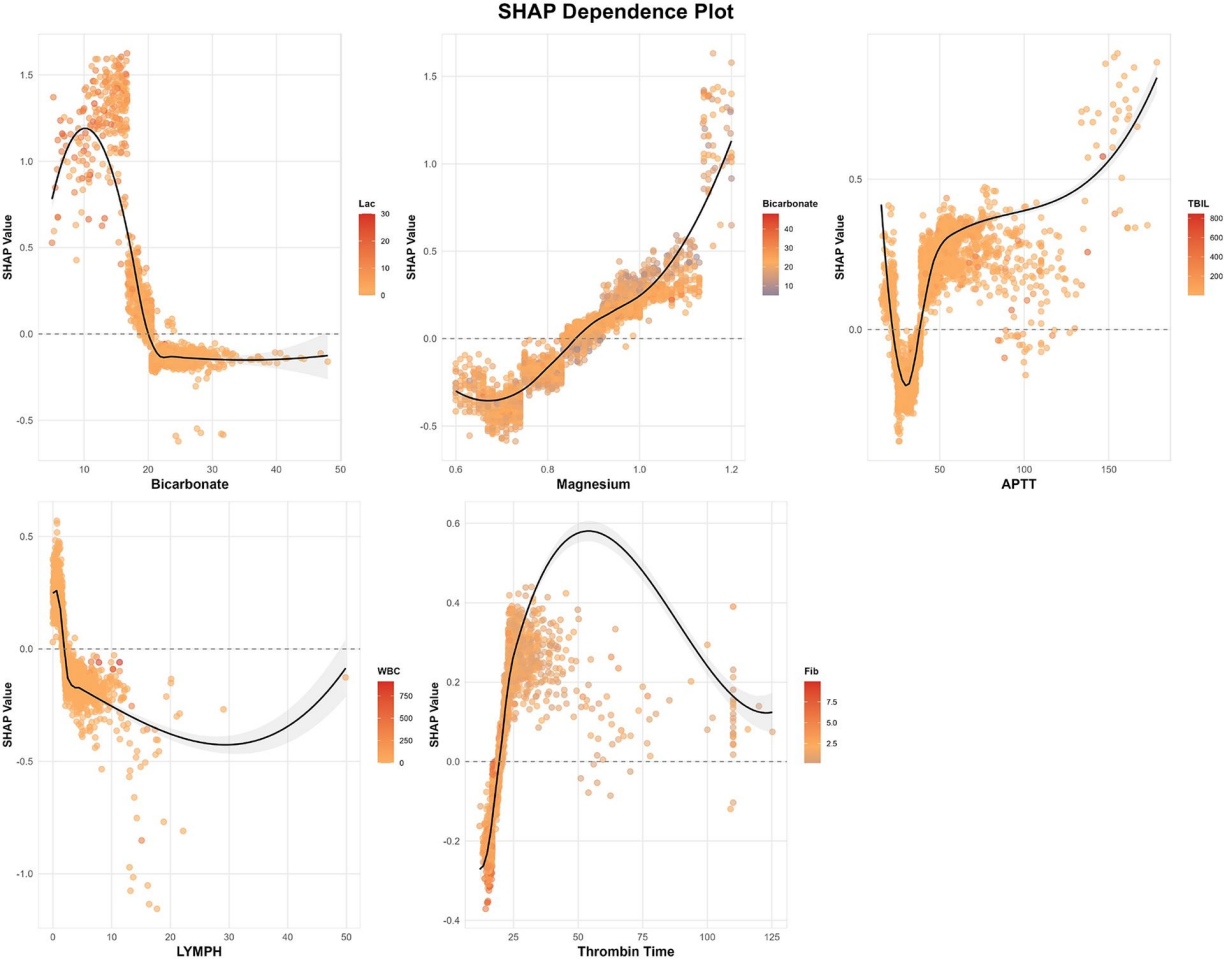
SHAP dependence plots for the top five most important features in the XGBoost model. *X*-axis: The feature value using clinically interpretable names with standardized units. *Y*-axis: "SHAP Value", where positive values indicate increased AKI risk and negative values indicate decreased risk. The black line represents a LOESS smooth, illustrating the nonlinear relationship between each feature and AKI risk. A horizontal dashed gray line is drawn at SHAP = 0 to visually separate risk-increasing (positive) from risk-decreasing (negative) contributions.

**Figure 7:**
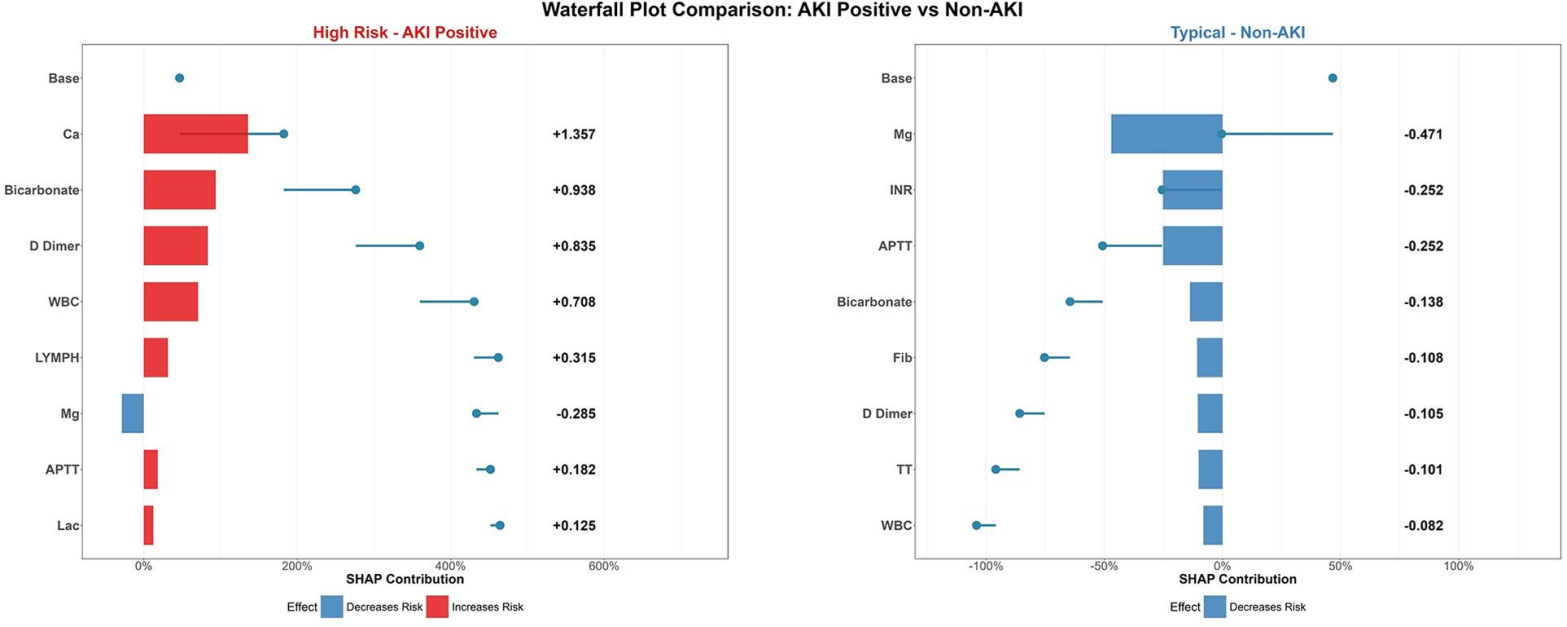
Individual SHAP explanations: waterfall plots of two representative cases. Left: High-risk AKI-positive patient — Ca, bicarbonate, and D-dimer are the primary risk drivers; Mg provides a protective effect. Right: Typical non-AKI patient — all features show protective effects (blue bars), with Mg contributing the most. Each plot starts from the baseline and shows how features additively shift the prediction toward higher (red) or lower (blue) risk.

To explore the directionality, threshold effects, and nonlinear relationships of the top predictors, we generated SHAP dependence plots for the five most important features (Figure 6). For bicarbonate, a clear threshold was observed at approximately 20 mmol/L: below this level, the SHAP value gradually becomes positive (indicating higher AKI risk), while above 20 mmol/L the effect tends to stabilize near zero (Figure 6). Additionally, to enhance understanding of the model's decision process at the individual level, we conducted a detailed interpretability analysis for two representative samples, as shown in Figure 7. Case 1 (left panel) — a high-risk AKI-positive patient — was primarily driven by calcium, bicarbonate and D-dimer as the main risk-increasing factors, while Mg provided a protective contribution. Case 2 (right panel) — a typical non-AKI patient — showed all features contributing in the protective direction, with Mg providing the largest negative contribution. These contrasting cases demonstrate that the same features can exert opposite effects depending on the patient's specific laboratory values, highlighting the clinical utility of SHAP waterfall plots for interpreting individual risk profiles. By visualizing the SHAP values for these samples, we can discern the direction and magnitude of each feature's impact on the model's risk assessment for these specific instances.

## 4 Discussion

Based on the PIC database, this study systematically constructed and compared five machine learning models for assessing AKI risk in critically ill children. The XGBoost model demonstrated acceptable discriminative ability. By combining LASSO regression and the Boruta algorithm for feature selection and employing SHAP analysis for model interpretation, we not only developed a model with acceptable risk stratification performance but also enhanced its comprehensibility and clinical applicability.

In the field of pediatric critical care medicine, the early identification and intervention of AKI remain a prominent clinical challenge. Although several studies have been dedicated to developing AKI prediction models (21–23), the applicability and predictive efficacy of these models in pediatric populations, particularly in critically ill children who exhibit significant physiological and pathophysiological heterogeneity, are limited. Furthermore, while some researchers have attempted to build AKI prediction tools specifically for pediatric critically ill patients, studies systematically applying and optimizing multiple machine learning algorithms for model construction remain relatively scarce (24–26). This study focused on a specific population—critically ill children. The constructed model incorporated multiple categories of features such as demographic information, laboratory indicators, medication use, and comorbidities, making it more aligned with the multifactorial pathophysiology of AKI in children. Compared to previous studies, the strategy of “dual-algorithm refined selection” (LASSO regression combined with Boruta algorithm) that we adopted retained features identified by both methods, effectively balancing the strengths of shrinkage estimation and nonlinear interaction detection, thereby enhancing the model's generalization ability. Our research has revealed that the incidence of acute kidney injury (AKI) among patients in the pediatric intensive care unit (PICU) is relatively high (46.6%), which is consistent with the reports from other studies (5, 16).

Recently, several studies have developed prediction models for pediatric AKI. Deng et al. used XGBoost to predict adverse outcomes within 30 days for children with diagnosed AKI, reporting an AUC of 0.810 (13). Sandokji et al. constructed a time-updated logistic regression model with 10 features for hospitalized children, and the internal validation AUC was 0.82 (14). In contrast, our XGBoost model achieved an AUC of 0.6838 in internal validation, which is lower than these recent reports. This difference may be attributed to the following factors: we used static predictors (the first 24 h after ICU admission) rather than time-series or time-updated data. Nevertheless, our study provides SHAP-based explanations for the interpretability of the XGBoost model in the general PICU population, offering interpretable risk explanations even with moderate discriminative performance. The DCA further supported the potential clinical usefulness of the XGBoost model: net benefit was positive across a clinically relevant range of threshold probabilities. This suggests that even with modest discriminative ability, the model could assist in early AKI risk stratification to guide monitoring or preventive interventions.

In the model comparison, ensemble learning algorithms (Random Forest, XGBoost, LightGBM) overall outperformed traditional Logistic Regression and SVM on the validation set, which is consistent with the findings of most medical prediction model studies in recent years (21, 27). However, we observed marked overfitting: Random Forest and LightGBM dropped from near-perfect training AUCs (1.000 and 0.9571, respectively) to approximately 0.67–0.68 on the validation set, indicating a clear risk of overfitting. XGBoost showed a smaller drop, suggesting relatively better generalization. SVM also exhibited overfitting. Notably, Logistic Regression had the smallest performance decline and demonstrated the best stability, but its calibration was suboptimal by quantitative metrics (HL *p* = 0.0207). Cross-validation results further supported these observations: the 5-fold cross-validated mean AUCs were 0.6749 (XGBoost), 0.6748 (Random Forest), 0.6704 (SVM), 0.6699 (LightGBM), and 0.6525 (Logistic Regression), with narrow standard deviations indicating stable performance across folds. Therefore, in scenarios where computational resources are limited or model transparency and calibration are paramount, Logistic Regression remains a valuable practical tool, whereas ensemble methods—particularly XGBoost—offer a reasonable trade-off between discrimination and overfitting risk. We acknowledge the severe overfitting of Random Forest and LightGBM; these models are not recommended for this dataset without stronger regularization, and future work should consider more aggressive regularization strategies.

This study confirmed the central role of several clinically relevant predictors in AKI risk assessment. SHAP analysis identified bicarbonate as the most influential predictor in the XGBoost model, contributing 13.74% of the total feature importance. Bicarbonate reflects acid-base balance, and its prominence supports the well-established role of metabolic acidosis in AKI pathogenesis: acidosis can exacerbate tubular injury, impair renal autoregulation, and promote inflammation (28, 29). Our SHAP dependence plot revealed a threshold effect for serum bicarbonate at approximately 20 mmol/L, below which AKI risk increased progressively. This finding aligns with the clinical definition of metabolic acidosis and supports the known pathophysiological role of acidosis in tubular injury and renal hypoperfusion. Magnesium ranked second; hypomagnesemia has been linked to endothelial dysfunction and increased oxidative stress, which may predispose to ischemic-reperfusion injury in the kidney (30). Activated partial thromboplastin time, lymphocyte count, and thrombin time completed the top five features. The high ranking of coagulation parameters (APTT, TT) and D-Dimer underscores the involvement of coagulation disorders and microvascular thrombosis in AKI development, particularly in the context of systemic inflammatory response syndrome or disseminated intravascular coagulation (31). Moreover, we observed an interaction between bicarbonate and thrombin time: the protective effect of higher bicarbonate levels was attenuated when thrombin time was prolonged, suggesting that coagulation disturbances may modify the renal response to acid–base imbalances. Lymphocyte count, as a marker of immune and inflammatory status, further highlights the role of systemic inflammation in renal injury. Notably, the top five features cumulatively contributed 56.77% of the overall importance, and the top ten features (bicarbonate, Mg, APTT, LYMPH, TT, calcium, white blood cell count, D-dimer, lactate, and INR) accounted for 88.37%. This concentration of risk-associated signal power within a core set of clinical variables enhances the model's interpretability and face validity, as the identified predictors align well with established pathophysiological mechanisms.

This study has several limitations. First, as a retrospective, single-center study using the PIC database, there are inherent biases such as missing data and selection bias. Although we excluded variables with a missing rate exceeding 20% and performed multiple imputation, residual biases may persist. Second, the lack of external validation limits the generalizability of our findings to other institutions or populations. It remains unclear how this model performs in other PICUs, in different geographical regions, or in newer cohorts. Although the PIC database is of great value, its single-center origin may affect the generalizability of the results. Third, this study used only static predictive variables from the first 24 h after admission and did not include time-series data (such as hourly urine output or dynamic creatinine changes), which could have provided earlier and more accurate warnings of AKI. Another limitation is the temporal overlap between predictor acquisition and AKI diagnosis. In the study cohort, some patients developed AKI within the first 24 h after admission, meaning that the outcome for these cases occurred during or before the prediction assessment window. Therefore, our model is more appropriately described as a tool for early detection of AKI rather than a purely prospective prediction model. Fourth, some potentially important variables (such as fluid balance and detailed medication doses) could not be continuously obtained due to incomplete data and were therefore not included. Fifth, although SHAP analysis improves model interpretability, the complex nonlinear relationships captured by XGBoost remain difficult to fully explain in simple clinical terms. Sixth, we acknowledge that performing feature selection exclusively on the training set may have contributed to the over-optimistic training performance observed for the ensemble methods, as this process may capture training-specific noise. To mitigate this risk, we prioritized validation set AUC and repeated cross-validation metrics for model evaluation. Additionally, the marked overfitting observed for Random Forest and LightGBM indicates that these algorithms are not suitable for this dataset without considerably stronger regularization.

Future research should focus on multicenter prospective validation, integration of dynamic physiological parameters, and application of deep learning methods to further enhance the timeliness, accuracy, and generalizability of the model.

## 5 Conclusion

In this study, we successfully developed and validated an early risk stratification tool based on the XGBoost machine learning model for assessing AKI risk in critically ill children. By systematically comparing five algorithms, we identified XGBoost as the model with acceptable generalization performance on the validation set, demonstrating reasonable discriminative ability and clinical applicability. The feature selection strategy combining LASSO regression and the Boruta algorithm provided a comprehensive set of clinically relevant predictors. SHAP analysis clarified the importance of key risk-associated factors—particularly bicarbonate, magnesium, coagulation parameters (APTT, TT), and lymphocyte count—offering valuable insights into the pathophysiological mechanisms underlying AKI, including acid-base imbalance, electrolyte disturbances, coagulopathy, and systemic inflammation. The concentration of risk-associated signal within a core set of variables enhances the model's interpretability and potential clinical utility. These findings may help guide early risk stratification, renal function protection, and targeted interventions in critically ill children, with the goal of improving treatment outcomes and quality of life. Future work will focus on external validation using multicenter, prospective data, incorporation of dynamic physiological parameters, and integration of this risk stratification tool into clinical decision support systems to enable precision and individualized management in pediatric critical care.

## Data Availability

The original contributions presented in the study are included in the article/Supplementary Material, further inquiries can be directed to the corresponding authors.
