# Supplementary figures and images for: Machine learning early risk assessment model for acute kidney injury in critically ill children: a retrospective cohort study
Source: Front Pediatr. 2026 Jul 9;14:1847661. doi: 10.3389/fped.2026.1847661 (PMC13391843; doi:10.3389/fped.2026.1847661)

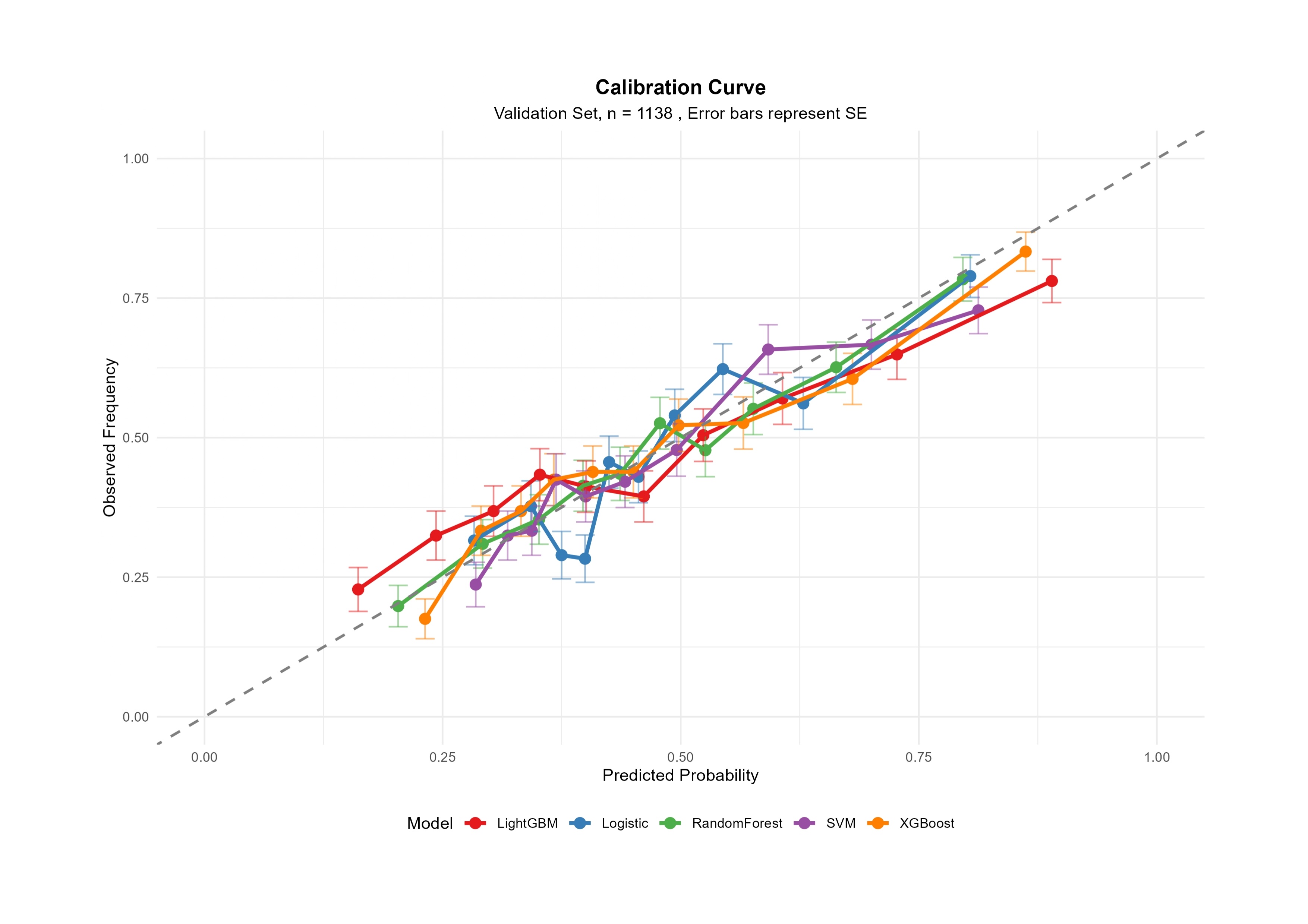

Supplement: Supplementary file 1 [file Image1.jpeg]

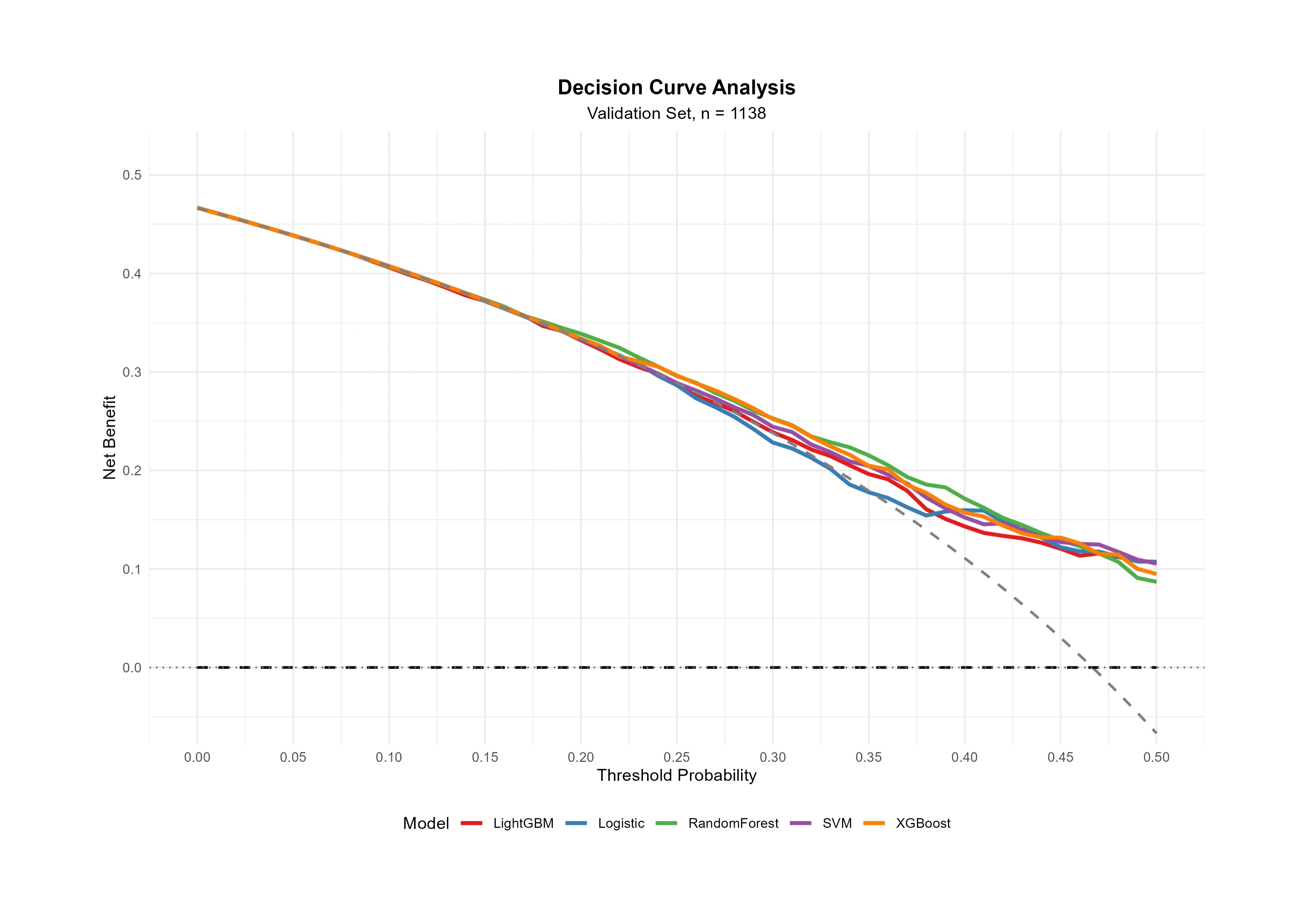

Supplement: Supplementary file 2 [file Image2.jpeg]
