# Supplementary material for: Machine learning early risk assessment model for acute kidney injury in critically ill children: a retrospective cohort study
Source: Front Pediatr. 2026 Jul 9;14:1847661. doi: 10.3389/fped.2026.1847661 (PMC13391843; doi:10.3389/fped.2026.1847661)
Supplement: Supplementary file 3 [file Supplementaryfile1.docx]

| Supplementary Table 1. Missing count and percentage by variable | | |  |
| --- | --- | --- | --- |
| **Variable** | **Missing Count** | **Missing Percentage** | **Retained in final model** |
| AG | 946 | 24.9% | No |
| Heart Rate | 922 | 24.27% | No |
| Respiratory Rate | 854 | 22.48% | No |
| Mg | 598 | 15.74% | Yes |
| TT | 556 | 14.64% | Yes |
| Fib | 543 | 14.29% | Yes |
| D-Dimer | 540 | 14.21% | Yes |
| CRP | 383 | 10.08% | Yes |
| GLU | 143 | 3.76% | Yes |
| PT | 112 | 2.95% | Yes |
| APTT | 111 | 2.92% | Yes |
| INR | 110 | 2.9% | Yes |
| TBIL | 85 | 2.24% | Yes |
| Ca | 33 | 0.87% | Yes |
| NEUT | 25 | 0.66% | Yes |
| LYMPH | 24 | 0.63% | Yes |
| Lac | 9 | 0.24% | Yes |
| ALB | 8 | 0.21% | Yes |
| Bicarbonate | 3 | 0.08% | Yes |
| RDW | 1 | 0.03% | Yes |
| Primary disease | 0 | 0% | Yes |
| AKI | 0 | 0% | Yes |
| Gender | 0 | 0% | Yes |
| PLT | 0 | 0% | Yes |
| WBC | 0 | 0% | Yes |
| Hb | 0 | 0% | Yes |
| Na | 0 | 0% | Yes |
| K | 0 | 0% | Yes |
| CHD | 0 | 0% | Yes |
| CKD | 0 | 0% | Yes |
| Sepsis | 0 | 0% | Yes |
| Tumor | 0 | 0% | Yes |
| Used vasopressor | 0 | 0% | Yes |
| Age | 0 | 0% | Yes |
| RBC | 0 | 0% | Yes |
