# Supplementary material for: Machine learning early risk assessment model for acute kidney injury in critically ill children: a retrospective cohort study
Source: Front Pediatr. 2026 Jul 9;14:1847661. doi: 10.3389/fped.2026.1847661 (PMC13391843; doi:10.3389/fped.2026.1847661)
Supplement: Supplementary file 4 [file Supplementaryfile2.docx]

Supplementary Table 2. Sensitivity Analysis Results: Comparison of Original and Imputed Data; Imputation method: PMM for numeric, Polyreg for factor

| **Variable** | **Original Mean** | **Imputed Mean** | **Mean Difference** | **SMD** | ***P-value*** |
| --- | --- | --- | --- | --- | --- |
| RDW | 14.4899 | 14.4898 | 0.0000 | 0.0000 | *0.9993* |
| NEUT | 7.2272 | 7.2427 | 0.0155 | 0.0028 | *0.864* |
| TBIL | 21.5675 | 21.4149 | -0.1526 | -0.0036 | *0.8282* |
| Ca | 2.2026 | 2.2027 | 0.0002 | 0.0007 | *0.9645* |
| ALB | 37.2575 | 37.2489 | -0.0085 | -0.0013 | *0.9354* |
| Bicarbonate | 22.2055 | 22.1982 | -0.0073 | -0.0019 | *0.9061* |
| CRP | 34.1424 | 33.6800 | -0.4625 | -0.0111 | *0.5218* |
| Lac | 2.5237 | 2.5305 | 0.0069 | 0.0029 | *0.857* |
| Fib | 2.3186 | 2.2990 | -0.0196 | -0.0177 | *0.322* |
| GLU | 8.6547 | 8.6292 | -0.0255 | -0.0060 | *0.7183* |
| D-Dimer | 3.3735 | 3.3937 | 0.0202 | 0.0040 | *0.8255* |
| INR | 1.2591 | 1.2589 | -0.0003 | -0.0005 | *0.9772* |
| PT | 15.3582 | 15.3570 | -0.0012 | -0.0001 | *0.9931* |
| TT | 20.9986 | 21.1823 | 0.1837 | 0.0179 | *0.3191* |
| APTT | 41.1926 | 41.1976 | 0.0050 | 0.0002 | *0.9884* |
| Mg | 0.8655 | 0.8686 | 0.0031 | 0.0243 | *0.2395* |
| LYMPH | 2.6482 | 2.6653 | 0.0171 | 0.0071 | *0.659* |

Note: * indicates statistical significance (p < 0.05)
