# Supplementary material for: Machine learning early risk assessment model for acute kidney injury in critically ill children: a retrospective cohort study
Source: Front Pediatr. 2026 Jul 9;14:1847661. doi: 10.3389/fped.2026.1847661 (PMC13391843; doi:10.3389/fped.2026.1847661)
Supplement: Supplementary file 5 [file Supplementaryfile3.docx]

Supplementary Table 3. Hyperparameter tuning strategy

| **Model** | **Tuning Method** | **Evaluation Metric** | **Early Stopping** | **CV Folds** | **Random Search** |
| --- | --- | --- | --- | --- | --- |
| Logistic (Elastic Net) | Grid search + 5-fold CV | AUC | No | 5 | No |
| Random Forest | Grid search + 5-fold CV | AUC (approx.) | No | 5 | No |
| XGBoost | Grid search + 5-fold CV (xgb.cv) | AUC | No | 5 | No |
| LightGBM | Grid search + 5-fold CV (lgb.cv) | AUC | Yes (10 rounds) | 5 | No |
| SVM (RBF) | Grid search + 5-fold CV (manual loop) | AUC | No | 5 | No |
| Note: CV = cross-validation; AUC = area under the ROC curve.  Supplementary Table 4. hyperparameter_search_ranges   \| **Model** \| **Parameter** \| ***Search Range*** \| **Selected Best Value** \| \| --- \| --- \| --- \| --- \| \| Logistic (Elastic Net) \| alpha \| *{0, 0.2, 0.5, 0.8, 1}* \| 0 \| \|  \| lambda \| *Automatically selected by cv.glmnet* \| 0.00869 \| \| Random Forest \| ntree (number of trees) \| *{200, 300, 500}* \| 300 \| \|  \| mtry (features per split) \| *{sqrt(p), p/3, p/2}* \| 3 \| \| XGBoost \| max_depth \| *{3, 4, 5}* \| 3 \| \|  \| eta (learning rate) \| *{0.01, 0.05, 0.1}* \| 0.05 \| \|  \| nrounds \| *{100, 150, 200} (selected by CV)* \| 150 \| \| LightGBM \| num_leaves \| *{15, 20, 31}* \| 15 \| \|  \| max_depth \| *{3, 4, 5}* \| 4 \| \|  \| learning_rate \| *{0.01, 0.05, 0.1}* \| 0.05 \| \| SVM (RBF) \| cost \| *{0.1, 0.5, 1, 2, 5}* \| 1 \| \|  \| gamma \| *{0.01, 0.05, 0.1, 0.5, 1}* \| 0.05 \|   Note: Random seed=123 | | | | | |

Supplementary Table 5. Best parameters summary

| **Model** | **Best Hyperparameters** | **Cross-Validation AUC** |
| --- | --- | --- |
| Logistic (Elastic Net) | alpha = 0, lambda = 0.00869 | 0.6525 |
| Random Forest | ntree = 300, mtry = 3 | 0.6748 |
| XGBoost | max_depth = 3, eta = 0.05, nrounds = 150 | 0.6749 |
| LightGBM | num_leaves = 15, max_depth = 4, learning_rate = 0.05 | 0.6699 |
| SVM (RBF) | cost = 1, gamma = 0.05 | 0.6704 |
| Note: Best parameters were selected using 5-fold cross-validation with AUC as evaluation metric. | | |
