# Supplementary material for: Machine learning early risk assessment model for acute kidney injury in critically ill children: a retrospective cohort study
Source: Front Pediatr. 2026 Jul 9;14:1847661. doi: 10.3389/fped.2026.1847661 (PMC13391843; doi:10.3389/fped.2026.1847661)
Supplement: Supplementary file 6 [file Supplementaryfile4.docx]

Supplementary Table 6. Calibration metrics

| **Model** | **Brier Score** | **Hosmer-Lemeshow χ²** | **HL p-value** | **Calibration Intercept** | **Calibration Slope** | **ECE** | **CITL** |
| --- | --- | --- | --- | --- | --- | --- | --- |
| Logistic | 0.2293 | 18.07 | 0.0207 | -0.0511 | 0.8963 | 0.0532 | 0.009 |
| RandomForest | 0.2236 | 3.53 | 0.8970 | -0.0272 | 0.9342 | 0.0213 | 0.004 |
| XGBoost | 0.2216 | 10.62 | 0.2243 | -0.0301 | 0.8789 | 0.0404 | 0.002 |
| LightGBM | 0.2285 | 33.61 | 0.0000 | -0.0595 | 0.6499 | 0.0616 | 0.000 |
| SVM | 0.2267 | 8.58 | 0.3793 | -0.0133 | 0.8767 | 0.0348 | 0.009 |
| Note: Brier score (lower is better); HL p-value > 0.05 indicates good calibration; ECE: Expected calibration error; CITL: Calibration-in-the-large | | | | | | | |
