# Supplementary material for: Machine learning early risk assessment model for acute kidney injury in critically ill children: a retrospective cohort study
Source: Front Pediatr. 2026 Jul 9;14:1847661. doi: 10.3389/fped.2026.1847661 (PMC13391843; doi:10.3389/fped.2026.1847661)
Supplement: Supplementary file 7 [file Supplementaryfile5.docx]

Supplementary Table 7a. Variables selected only by LASSO

| **Number** | **Variable** |
| --- | --- |
| 1 | WBC |
| 2 | PLT |
| 3 | Hb |
| 4 | LYMPH |
| 5 | TBIL |
| 6 | Ca |
| 7 | Mg |
| 8 | K |
| 9 | Bicarbonate |
| 10 | Lac |
| 11 | D_Dimer |
| 12 | Fib |
| 13 | INR |
| 14 | TT |
| 15 | APTT |
| 16 | CHD |
| 17 | Tumor |
| 18 | Used_vasopressor |
| 19 | Primary_disease |

Supplementary Table 7b. Variables selected only by Boruta

| **Number** | **Variable** |
| --- | --- |
| 1 | WBC |
| 2 | PLT |
| 3 | CRP |
| 4 | LYMPH |
| 5 | TBIL |
| 6 | Ca |
| 7 | Mg |
| 8 | K |
| 9 | Bicarbonate |
| 10 | Lac |
| 11 | D_Dimer |
| 12 | Fib |
| 13 | INR |
| 14 | TT |
| 15 | APTT |
| 16 | PT |
| 17 | Age |
| 18 | Used_vasopressor |
| 19 | Primary_disease |
| 20 | Na |
| 21 | GLU |
| 22 | ALB |
| 23 | NEUT |
| 24 | RDW |

Supplementary Table 7c: Intersection - These are the final predictor variables used in all models.

| **Number** | **Variable** |
| --- | --- |
| 1 | WBC |
| 2 | PLT |
| 3 | LYMPH |
| 4 | TBIL |
| 5 | Ca |
| 6 | Mg |
| 7 | K |
| 8 | Bicarbonate |
| 9 | Lac |
| 10 | D_Dimer |
| 11 | Fib |
| 12 | INR |
| 13 | TT |
| 14 | APTT |
